# Intestinal *ex vivo* organoid culture reveals altered programmed crypt stem cells in patients with celiac disease

**DOI:** 10.1038/s41598-020-60521-5

**Published:** 2020-02-26

**Authors:** Walburga Dieterich, Markus F. Neurath, Yurdagül Zopf

**Affiliations:** 1 Department of Medicine 1, Friedrich-Alexander-Universität Erlangen-Nürnberg, Erlangen, Germany; 2 Hector Center of Excellence for Nutrition, Exercise, and Sports, Friedrich-Alexander-Universität Erlangen-Nürnberg, Erlangen, Germany

**Keywords:** Gastrointestinal diseases, Gastrointestinal models

## Abstract

The *ex vivo* generation of gastrointestinal organoids from crypt stem cells opens up the possibility of new research approaches investigating gastrointestinal diseases. We used this technology to study differences between healthy controls and patients with celiac disease (CD). We noticed distinct dissimilarities in the phenotypes of organoids between our study groups and found considerable variations in their gene expression. Extracellular matrix genes involved in epithelial-mesenchymal transition are expressed most differently. In addition, we demonstrated epigenetic modifications that might be responsible for the different organoid gene expression thus accounting for a deranged crypt/villus axis development in CD. The organoids have proven valuable to demonstrate fundamental differences in duodenal derived organoids between healthy controls and patients with CD and thus are a suitable tool to gain new insights in pathogenesis of CD.

## Introduction

The human gastrointestinal tract is a highly complex organ and responsible for the adequate transport and digestion of foods as well as the resorption of nutrients, vitamins, minerals, and electrolytes. Caused by the multifaceted tasks and the constant confrontation with allergens and gut microbiota, an adequate gut barrier and a well-balanced gut associated immune system are required as a prerequisite and warranty for optimal functioning and intestinal homeostasis. Due to the complexity of the gut, the establishing of a functioning *ex vivo* intestinal model system is highly challenging and has long been the subject of intensive research. Organoids from murine intestine have been well established as *ex vivo* model for several years. However, it has become clear that isolation, proliferation, and cultivation of human gastrointestinal organoids are much more demanding. Recently, the propagation of adult stem cells to *ex vivo* organoid cultures from a wide panel of human gastrointestinal epithelial cells have successfully been established^1–3^. Interestingly, the long-term culture of human organoids has already been used to demonstrate tissue-specific and age-related accumulation of genetic mutations in adult stem cells^4^, and *ex vivo* organoids from ovarian cancers reflect the histological and genomic diversity of the underlying different types of carcinoma as well as intra- and interpatient heterogeneity^5^. Because the organoids possess many functional and structural characteristics of the original tissue and retain their tumorigenic potential, this technology has now opened completely new perspectives in human research. Organoids generated from individual patients offer the future to study and identify molecular mechanisms in pathophysiology, in transformation and progression of tumor growth, screening and prediction of response and sensitivity to chemotherapeutic drugs, and ultimately the possibility of personalized therapy^6,7^.

Our aim was to isolate human duodenal crypt stem cells and cultivate intestinal organoids from healthy controls and patients with celiac disease (CD). CD is an enteropathy, triggered by the ingestion of dietary gluten. Genetically predisposed patients, who possess the major histocompatibility markers DQ2 or DQ8, develop a mucosal damage characterized by villi atrophy and crypt hyperplasia mainly in duodenal and jejunal mucosa^8,9^. Gluten ingestion causes the inappropriate activation of CD4+ T-cells, which recognize gluten peptides presented by DQ2 or DQ8^10^. Tissue transglutaminase (TG2) has been identified as autoantigen in CD^11^, and IgA antibodies against TG2 are highly sensitive and specific markers in diagnosis of CD^12,13^. Interestingly, TG2 is enriched in mucosa of celiac patients and able to deamidate gluten peptides, rendering them more affine to DQ2 or DQ8, thus potentiating the immunoreaction^14^. Since about 30% of the population has the genetic predisposition to develop CD, but only a fraction of it actually develops it (estimated prevalence of 1% in Europe and US), further external factors in the development of the disease are discussed^15,16^. Recently, Freire *et al*. have successfully used gut organoids from patients with CD to show significantly altered gene expression concerning the gut barrier, innate immune response, and stem cell function compared to organoids from healthy controls. Furthermore, monolayers derived from these organoids demonstrated increased intestinal permeability and different secretory cytokine patterns between CD and controls^17^. Our study aimed to identify major basic differences between organoids from healthy subjects and patients with CD in order to yield further insights in the pathogenesis of CD that is characterized by a deranged crypt/villus axis development in CD.

## Results

### Basic features of organoids generated of patients with CD and healthy controls

**Figure 1:**
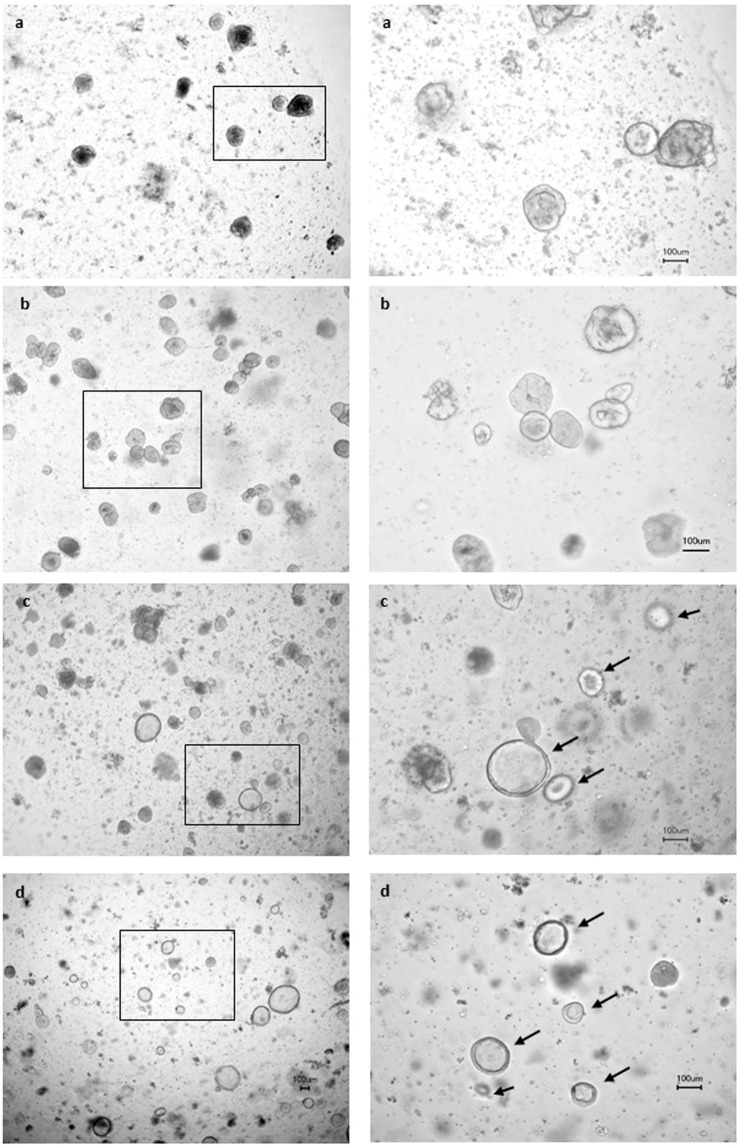
Organoids from intestinal biopsies of patients with CD and healthy controls. Organoids of patients with CD (**a**,**b**) showed a compact dense appearance, whereas organoids of healthy individuals (**c,d**) displayed balloon like features (see arrows). Magnification left column 40×; right column 100×. Magnified sections are framed.

We cultured organoids from duodenal biopsies to compare basic features between groups. Five to seven day cultivation of crypt units yielded organoids with 50–200 µm diameters. There was no apparent difference in numbers or size of organoids from patients with CD or healthy individuals. Furthermore, there were no variations in 40 housekeeping genes and genes involved in cell cycle and apoptosis thus excluding crucial alterations in basic metabolisms. (Corresponding RNA data are available in Supplemental Table 1) However, the rounded organoids from patients with CD showed a compact dense appearance and this clearly distinguished them from the rather tender-looking balloon like features of organoids that were obtained from healthy persons. This phenotypical difference was still maintained after splitting of organoids (Fig. 1).

### Differentiation of organoids derived from duodenal biopsies to reflect the *in vivo* situation as realistic as possible

Organoid cultures were grown for approximately two weeks until about 70% of organoids reached at least a size of 150–200 µm before the culture medium was changed against differentiation medium. Our previous studies have shown that changing of culture conditions with deprivation of growth factors, but supplementation of γ-secretase inhibitor DAPT caused differentiation of organoids already after two days. The differentiation was confirmed with organoids from two patients with non-celiac glutensensitivity by decreased mRNA expression of stem cell marker LGR5+ (0,21 and 0,006 fold) and an increase in goblet cell marker mucin 2 (3,72 and 62,04 fold) (Supplemental Table 2).

### Gene expression of organoids using the NanoString human myeloid innate immunity V2 panel

**Figure 2:**
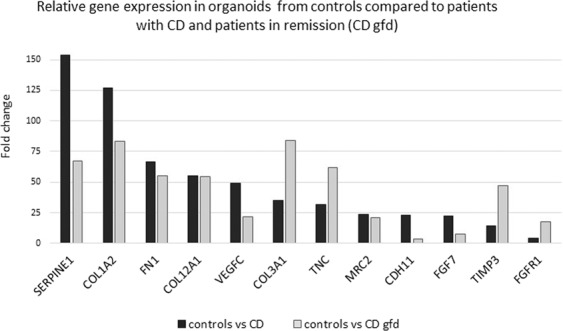
Gene expression analyzed by NanoString human myeloid innate immunity V2 array. Fold change expression. Shown are the 12 most differentially expressed genes from RNA pools of controls (n = 5) compared to patients with CD (n = 2) and CD in remission (CD gfd; n = 5, respectively. Complete gene expression and NM data are shown in Supplemental Table 1.

The differences in phenotypic appearance of organoids from patients with CD and controls suggested variations in cell adhesion, cell cycle, or differentiation. Therefore, the myeloid innate immunity V2 panel was chosen for RNA analysis. The NanoString analysis revealed great differences between the organoids derived from controls (n = 5) and organoids from patients with CD (n = 2). Interestingly, only minor alterations were noticed between patients with active CD (n = 2) and patients under gluten-free diet (gfd; n = 5). Most interesting is the different regulation of genes concerning the extracellular matrices (ECM). Collagen 1 alpha chain 2 (COL1A2), collagen 3 alpha chain 1 (COL3A1), and collagen 12 alpha chain 1 (COL12A1), fibronectin (FN1), tenascin C (TNC), and tissue inhibitor of metalloprotease 3 (TIMP3) showed a considerably diminished expression in patients with CD compared to healthy controls. In addition, cadherin 11 (CDH11), vascular endothelial growth factor C (VEGFC), mannose receptor C type 2 (MRC2), serpine peptidase inhibitor (SERPINE1), fibroblast growth factor 7 (FGF7), and fibroblast growth factor receptor 1 (FGFR1) were clearly detected in healthy controls whereas patients with CD displayed only minor expression of these genes (Fig. 2). Complete gene expression, NM data, and fold change are shown in Supplemental Table 1.

The data derived from the NanoString analysis were confirmed exemplary by quantitative RT-PCR. cDNA pools from five healthy controls and five celiac patients on a gfd were checked for expression of COL1A2, COl12A1, FN1, and SERPINE1. As expected, the pooled cDNA of healthy controls revealed increased ECM gene expression compared to celiac patients on gfd (Table 1).

**Table 1:** RT-PCR amplification of genes of interest to confirm RNA array data derived from NanoString analysis.

Target		Ctrl	Fold expression	Mean Cq
**Relative mRNA expression of selected genes of organoids**				
GAPDH	controls			24,69
GAPDH	CD gfd	*		22,08
COL1A2	controls		15,553	35,83
COL1A2	CD gfd	*	1,000	35,85
COL12A1	controls		100,525	28,33
COL12A1	CD gfd	*	1,000	32,37
FN1	controls		169,587	25,52
FN1	CD gfd	*	1,000	30,31
SERPINE1	controls		182,416	26,21
SERPINE1	CD gfd	*	1,000	31,11

Fold-change analysis was done with ΔΔC_t_ method with GAPDH for housekeeping gene. cDNA from organoids of healthy controls (n = 5) was pooled and gene expression correlated to basic expression of pooled cDNA of organoids derived from celiac patients on gfd (n = 5). Organoids of healthy controls showed clearly enhanced mRNA expression of COL1A2, COL12A1, FN1, and SERPINE1 compared to organoids of patients with CD on gfd.

**Figure 3:**
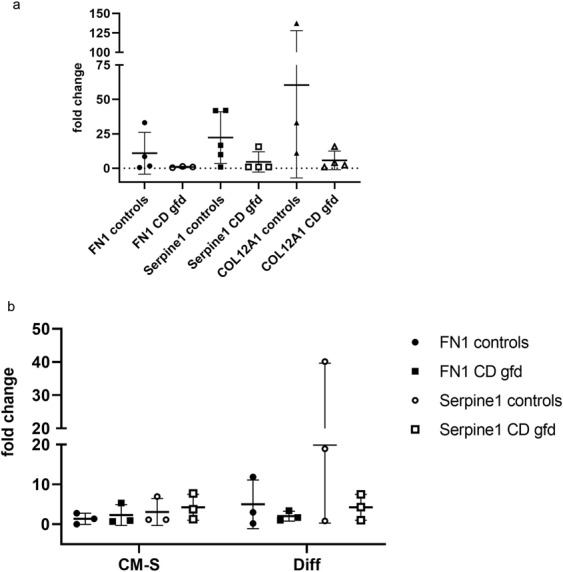
Relative mRNA expression of organoids from healthy controls compared to organoids from patients with CD on gfd. Scatter dot blot; line at mean with SD. Real-time PCR was performed for expression of FN1, SERPINE1, and COL12A1, and fold-change analysis was done with ΔΔC_t_ method. GAPDH was used for housekeeping gene and values correlated to basic expression of sample from CD patient on gfd. (**a**) Organoids derived from duodenal biopsies of healthy controls (n = 3–5) showed clearly higher expression of FN1, SERPINE1, and COL12A1 than samples from patients with CD on gfd (n = 3–5). Single data are missing caused by low cDNA concentrations. (**b**) No different gene expression was noticed in non-differentiated organoids cultured in CM-S. Differentiation for two days resulted in increased levels of FN1 and SERPINE1 in organoids from controls (n = 3) compared to CD gfd (n = 3).

To exclude the possibility that data are biased by a single patient with disorders in ECM, we exemplary measured samples of single individuals for expression of FN1, SERPINE1, and COL12A1. We found that three out of five healthy controls showed elevated expression for FN1 and COL12A1, and four out of five controls showed increased levels for SERPINE1. Some data are missing due to small amounts of available cDNA. Celiac patients on gfd had no increased levels under gfd for FN1 and only one patient had increased levels for SERPINE1 and COL12A1. Although mean values differ clearly between controls and patients they do not reach significance due to the small number of cases (Fig. 3a; Supplement Table 3).

### ECM gene expression in proliferating non-differentiated organoids does not vary between groups

Cryo conserved organoids from three healthy individuals and three celiac patients on long-term gfd were thawed and cultured in CM-S medium till organoids reached an average diameter 150 µm. Pooled RNA from both groups showed no difference in ECM gene expression of COL12A1, FN1, or SERPINE1. However, changing culture condition for two days to differentiation yielded increased levels of these ECM genes in organoids from healthy controls compared to organoids from patients with CD on gfd (Supplemental Table 4).

However, mean data do not reach the values as high as in former assay with fresh organoids. After examining the individual organoid gene expression it turned out that one of the healthy controls failed to express ECM genes FN1 and SERPINE1, and interestingly this subject showed activated lymph follicles in histopathology, and thus rather must be classified as sick person (Fig. 3b; Supplemental Table 5).

### Gene expression profile from whole intestinal biopsies

cDNAs from shock-frozen duodenal biopsies were available from three healthy controls, two patients with active CD, and five celiac patients on gfd. Interestingly, the amplification data for COL1A2, FN1, and SERPINE1 showed no main differences in the gene expression between all three groups. This was caused by a minor expression of the analyzed ECM genes from the tissue samples of healthy controls compared to *ex vivo* cultured organoids, and the expression values are as low as from tissue samples of patients with CD. (Supplemental Table 6).

### Determination of genes from intestinal organoids involved in epithelial to mesenchymal transition

**Figure 4:**
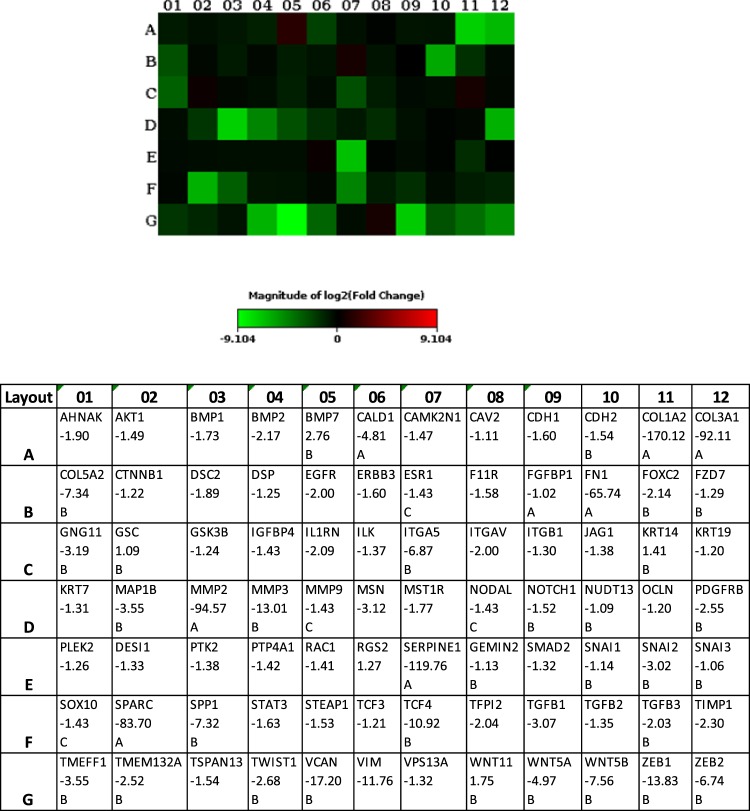
Gene expression of intestinal organoids generated from patients with CD (n = 3) and healthy controls (n = 7). Heat map to show fold changes in gene expression of organoids. Genes under-expressed in patients with CD versus controls are shown in green. The table provides the fold regulation data. Data analysis was performed using the webportal of Qiagen. Fold changes of genes were calculated with the ΔΔC_t_ method and shown in the corresponding table. Comment A means: The gene’s average threshold cycle is relatively high in one group but reasonably low in the other group. Therefore, actual fold-change value is at least as large as the calculated and reported fold-change result. B: The relative gene expression level is relatively low, in both groups. C: Gene expression is very low, making this fold-change result un-interpretable.

84 genes involved in epithelial to mesenchymal transition were tested by RT^2^ Profiler PCR Array. The array confirmed the remarkable downregulation of ECM genes COL1A2, COL3A1, FN1, MMP2, SERPINE1, SPARC (osteonectin), and also distinct reduced expression of COL5A2, MMP3, and VCAN (versican), in organoids from patients with CD (n = 3) compared to healthy individuals (n = 7) (Fig. 4, Supplemental Table 7).

We could only note minor differences in gene expression between patients with active CD (pool of n = 3) and patients in remission under gfd (n = 5) (Data available in Supplemental Fig. 1 and Supplemental Table 7).

### Epigenetic chromatin remodeling to study differences in gene remodeling

The RT^2^ Profiler PCR Array “human epigenetic chromatin remodeling array” (Qiagen, Germany) allows determination of 84 genes, which are involved in chromatin remodeling. Patients with active CD showed a differentiated chromatin regulation with a clear upregulation of bromodomain containing 7 (BRD7), inhibitor of growth family member 4 (ING4), and methyl CpG binding protein 2 (MECP2), but decreased expression of chromobox homolog 4 and 5 (CBX4, CBX5), chromodomain helicase DNA binding protein 3, 6, and 7 (CHD3, CHD6, CHD7) (Supplemental Fig. 2).

## Discussion

The development, culture, propagation and long-term storage of organoids allow functional *ex vivo* studies with intestinal cells and combine the big advantage of easy accessible samples with the possibility to perform patient-based examinations^1^. Since the organoids retain their structural and functional properties from the original tissue this new research area is particularly interesting for the establishment of new tumor models^6,18^. When we established *ex vivo* organoid cultures from duodenal tissue of patients with CD and healthy controls, we noted distinct differences in phenotypes of organoids. Our aim was to identify the underlying elementary differences. This is of special interest since crypt stem cells are the origin of up to 300 cells the day, which differentiate along their apical migration to villus tip, where they are replaced every 3–5 days^19–21^. Interestingly, we detected genes involved in regulation of extracellular matrix (ECM) that were expressed very differentially, with high expression in organoids from healthy controls compared to only marginal levels in organoids from patients with CD. Organoids from patients with active CD and patients in remission displayed almost identical low RNA expression patterns, although duodenal samples from patients in remission showed no inflammation at all and thus rather resembled healthy tissue. We therefore postulate a fundamental difference in the genetic expression pattern between adult intestinal stem cells from individuals with healthy gut and patients with CD independent on disease stage.

Middendorp *et al*. have already demonstrated that region-specific gene expression is highly stable in long-term organoid cultures. The functional fate of adult intestinal stem cells is intrinsically programmed and stem cells display a regional gene expression profile that is independent of extracellular signal^22^. In our study, we noticed a different regulation of several genes involved in chromatin remodeling between organoids from healthy controls and celiac patients indicating epigenetic factors that may influence gene expression. Most strikingly was the upregulation of bromodomain containing protein 7 (BRD7; 8,2 fold), and the inhibitor of growth 4 (ING4; 3,5 fold) in patients with CD. BRD7 is associated with tumor progression in prostate cancer^23^, but upregulation of BRD7 also protects from excessive weight gain and hyperglycemia at least in mice studies^24^. ING members are involved in cell proliferation, apoptosis, senescence, and DNA replication and repair. ING4 is mainly suppressed in various tumors and coming along with poor prognosis in melanoma, hepatocellular, gastric and breast tumors. (For review see^25^) Furthermore, it has been shown that the expression of chromodomain helicase DNA binding proteins (CDH7) positively correlates with the growth rate of stem cells and their ability to proliferate^26^. Interestingly, CDH7 was downregulated in our patients with CD (−2.0 fold) compared to healthy controls. It is important to bear in mind that we examined organoids from healthy individuals and patients with CD, but without tumors. In our organoids from CD patients, several other genes involved in chromatin remodeling, e.g. MECP2, CBX 4 and 5, and CHD6, are differentially expressed. These data further emphasize a basic difference in stem cell propagation between organoids from CD patients and healthy subjects. Since epigenetic modifications confer cell-specific gene expression this topic is of special interest for further studies in CD pathogenesis. It will be particularly exiting to investigate whether individuals with increased risk for CD, e.g. first-degree relatives of patients with CD or patients with potential CD, i.e. increased celiac specific serum antibodies but without mucosal damage, exhibit typical epigenetic modifications that might predict the outcome of active CD.

Remarkably, we did not detect any differences in ECM gene expression of native whole intestinal biopsy samples derived from healthy individuals compared to celiac patients. Thus, the alteration in ECM gene expression was restricted to isolated *ex vivo* cultured organoids and mainly due to the strong expression of the ECM genes in organoids of healthy controls. This discrepancy between the data from organoids and biopsies can be explained by the fact that organoids consist mainly of stem cells, whereas intestinal biopsies largely contain differentiated epithelial cells. In this context, another research group isolated crypt areas from intestinal biopsies from five patients with active CD and five controls by laser capture microscopy to enrich the relative numbers of stem cells in their assay. They showed that some pathways in stem cell differentiation and proliferation are altered in CD, e.g. downregulation of hedgehog and WNT signaling pathway, and they already described a change in epithelial-mesenchymal transition that might be critical in active CD^27^. Interestingly, they also demonstrated a downregulation of zinc finger/homeodomain protein ZEB2, a marker that is also distinctly lower expressed in our organoids from celiac patients. ZEB2 is described in combination with TGFß signaling pathway and repression of E-cadherin, which is associated with the epithelial functional complex and thus much important for cell layer formation (For review^28^).

Our data showed slightly elevated Stat3 levels in organoids from controls compared to CD patients and CD gfd (1,6 fold and 2,1 fold, respectively; Supplemental Table 7). It has been shown that Stat3 binds to and is necessary for the activation of the COL1A2 enhancer^29^ and the COL1A2 enhancer is suggested to be central for the expression of collagen type 1 in embryogenesis, but is also activated by physical injury^30^. It is well known that ECM components are able to bind a panel of growth factors and thus modulate their availability and activity. In this context, it was shown that PDGF A and B, a mitogen for mesenchymal cells, accumulate in the ECM^31^, and collagens Type I, II, III, IV, and VI were identified as ligands for the PDGF isoforms AA, BB, and AB. A local enrichment with a molar ratio of 3–4 PDGF molecules bound to collagen fibers was going along with a 1.5-3 fold stimulation of the proliferation of human fibroblast^32^. Furthermore, the binding of the profibrogenic cytokine oncostatin M and epithelial mitogen keratinocyte growth factor to collagens I, III, and VI, as well as accumulation of fibroblast growth factor with ECM components were described^33–35^. ECM also plays a crucial role in the interaction of cells and serves as a scaffold for the adhesion of lymphocytes. The interaction of lymphocytes with ECM is necessary to adhere and migrate to the right place where they are needed. This interaction is controlled by several factors and it was shown that e.g. stem cell factor enhanced the adhesion of mast cells to several ECM proteins up to 5-fold^36^. In this context, our data demonstrating the enhanced expression of ECM components in organoids derived from crypt stem cells of healthy subjects are of huge importance since the ECM yields the basis for the adhesion and enrichment of several growth factors, which are necessary to build up a well-functioning scaffold as a prerequisite for tissue homeostasis. Interestingly, TG2 that was identified as autoantigen in CD^11^ and is responsible for the enhanced immunological response to gluten peptides, is bound via integrins to ECM proteins and is highly enriched in the intestinal mucosa of patients with CD^14,37^. TG2 plays a decisive role in the crosslinking and stabilization of ECM components and in wound healing, but is also described in pathological conditions, e.g. fibrosis, tumor metastasis, atherosclerosis, neurodegenerative diseases^35,38^. Future studies must show to what extent the altered ECM gene expression in patients with CD has an influence on the binding and availability of TG2 in CD.

Recently, Freire *et al*. have used the organoid technology to demonstrate differences in genes related with gut barrier, innate immune reaction, and stem cell function. In addition, they described an altered growth behavior with lower average area of organoids from patients with CD compared to healthy controls^17^. This is in contrast to our observations. While our organoids of patients with CD displayed a clearly altered, more compact phenotype compared to organoids of healthy controls there was no difference in size of organoids. This discrepancy might be explained by different culture conditions or variations in concentrations of Wnt3, R-spondin, and noggin in conditioned L-WRN medium. In accordance with data from Freire *et al*., we also noticed no variations in expression of apoptotic genes in organoids from controls or patients with CD. However, the use of organoids allowed us to identify an altered expression of genes involved in ECM and epithelial-mesenchymal transition in patients with CD compared to healthy controls. Epigenetic modifications might be responsible for the different gene expression of the crypt stem cells and account for a deranged crypt/villus axis development in CD. In our analysis, we focused on the genes with greatest difference between the groups. Nevertheless, it should be borne in mind that even small alterations or epigenetic modifications might have significant effects on gene expression. The organoid model allows further research on molecular mechanisms in CD and it would be of interest to examine organoids from non-damaged intestinal areas of patients with CD, e.g. terminal ileum, or from patients with potential CD for changes in gene expression or epigenetic variations. Since the organoid retain original tissue and disease specific properties, they also offer the possibility to study altered gene expression in *ex vivo* organoids from patients with other gastrointestinal disorders, e.g. Crohn’s disease or ulcerative colitis. Despite the enormous potential of organoids, one must be aware for future research that the culture of organoids is partly an artificial system that is based on crypt stem cells. This aspect has to be taken into account when transferring directly the data derived from organoids to *in vivo* situations.

## Methods

### Patients

Patients were recruited in the outpatient clinic of the Department of Medicine 1, FAU Erlangen-Nürnberg, between 2017 and 2018. Biopsy samples were obtained from overall seven healthy controls (5 females, 2 males, mean age 55–74 years) who underwent gastroscopy for preventive medical checkup, three patients with active CD (3 females, age 18, 19, and 55 years), and five celiac patients under remission (4 females, 1 male, mean age 21–39 years). The study was approved by ethics committee of FAU (Ethikkommission der Friedrich-Alexander-Universität Erlangen-Nürnberg, ethikkommission@fau.de, AZ 60_17B). All research was performed in accordance with the relevant guidelines and regulations, and informed consent was obtained from all participants. ClinicalTrials.gov ID: NCT03256266.

### Organoids

Two to three duodenal biopsies per individual were taken with standard endoscopic forceps during routine gastroduodenoscopy and placed in ice-cold 5 ml cell basal medium (DMEM/F12, 15 mM HEPES (Gibco, Germany), 2mM L-glutamine (Gibco, Germany), 100 u/ml penicillin, and 0,1 mg/ml streptomycin (Gibco, Germany) immediately. Crypt units were isolated according the protocol of VanDussen *et al*.^1^ with minor variations. The biopsy samples were washed twice with 5 ml cold basal medium (BM) before they were minced with a scalpel and enzymatically digested in 1 ml BM with collagenase (2 mg/ml, Sigma-Aldrich C9407) at 37 °C. The digest was pipetted every 5 minutes and microscopically controlled. When the tissue structure was mostly dispersed, usually after 20–25 minutes, the digest was filtered through a 100 µm strainer (Falcon, Germany) and strainer rinsed with additional 10 ml of BM. Crypt units were collected by low-threshold centrifugation (150–200 × g) for 5 minutes to separate from single cells and cell debris. In case of high impurity with cell debris the crypt cells were resuspended once more in 10 ml BM and pelleted at 150–200 g for 3 min. Cell pellets were resuspended in 1 ml BM, transferred to 2 ml vials and sedimented at 2000 × g for 5 min. The supernatant was discarded and crypts were carefully resuspended in 120 µl of ice-cold Matrigel matrix (Corning 35623) to enable three dimensional growths. Aliquots à 30 µl were settled in 24 well plates and plates were incubated in cell culture incubator at 37 °C, 5% carbondioxid for 10 minutes to allow the Matrigel to solidify. Afterwards, 250 µl cell culture medium enriched with supplements (CM-S) was added to each well and replaced every second day.

Organoids were used for assays or cryo conserved at −150 °C. Therefore, organoids were washed with ice cold BM to remove Matrigel and collected by centrifugation at 2000 × g for 5 min. Organoid pellets were suspended in 1 ml BM, 10% fecal calf serum (FCS, Biochrom, Germany), 10% dimethyl sulfoxide, slowly frozen to −80 °C in cryo freezing container (Nalgene, Germany), and transferred to −150 °C for long-term storage. For further research the cryo conserved organoids were quickly thawed at 37 °C, transferred to10 ml BM, centrifuged at 2000 × g for 5 min, plated with Matrigel and cultured in CM-S medium.

### Culture medium to maintain organoids (CM-S)

Mouse L-cells that express Wnt3a, R-spondin, and noggin, were commercially purchased (ATCC CRL-3276, Bio Tech Standards, Germany) and conditioned medium (L-WRN) was prepared according instructions and protocol of company and Miyoshi *et al*.^3^. Culture medium with supplements (CM-S) was prepared using 50% BM and 50% conditioned L-WRN medium, and further supplemented with 15% FCS, 1 mM N-acetyl-L-cysteine (Sigma, Germany), 1x N-2 supplements (Gibco, Germany), 1 × B-27®supplements (Gibco, Germany), 50 ng/ml epidermal growth factor, 10 mM nicotinamide (Sigma, Germany), 10 nM Leu15-gastrin I (Sigma, Germany), 500 nM A-83–01 (inhibitor for ALK4/5/7; Sigma, Germany), 10 µM SB202190 (p38 MAP kinase inhibitor; Sigma, Germany), and 10 µM Y-27632 (p160 ROCK inhibitor; Tocris, Germany) in accordance with the protocols of Sato *et al*., VanDussen *et al*., and Miyoshi *et al*.^1–3^. Organoids were cultured with 300 µl culture medium and medium changed every second or third day.

After five to seven days, when organoids have formed big-circled structures, they were isolated from Matrigel matrix and splitted. Therefore, organoids were washed once with ice-cold Dulbecco’s phosphate buffered saline (PBS; Gibco, Germany) and sheared by pipetting several times in 500 µl ice-cold PBS. Crushed organoids were transferred into 12 ml vials (Falcon, Germany), diluted with 10 ml PBS, pelleted by centrifugation at 150–200 × g and plated with Matrigel matrix as described above into 6–8 wells.

### Differentiation of organoids

For intestinal differentiation the culture medium was replaced by 300 µl of 90% BM and 10% L-WRN, 15% FCS, 10 µM Y-27632, and 5 µM γ-secretase inhibitor DAPT (Sigma, Germany) as described by VanDussen *et al*.^1^ and organoids were further cultivated for 2 days at 37 °C, 5% C0_2_.

### RNA-Isolation

Organoids were washed with cold PBS and incubated with 500 µl ice-cold cell recovery solutions (Corning, Germany) at 4 °C for 30 min. Organoids were sheared by several up and down pipetting, transferred into 12 ml vial (Falcon, Germany), diluted with 10 ml PBS and centrifuged at 200 × g. RNA isolation was performed with peqGOLD MicroSpin Total RNA Kit (VWR, Germany) according to manufacturer’s protocol.

In order to isolate RNA from intestinal tissue, the shock-frozen duodenal biopsies were homogenized with steel balls and the RNA was further isolated with RNeasy Plus Mini Kit (Qiagen, Germany). RNA concentration and purity were determined with Nanodrop 2000 spectrophotometer (ThermoScientific, Germany).

### RNA array

The isolated RNAs from organoids of five healthy individuals, two patients with active CD, and five patients in remission were pooled, respectively. For RNA gene expression analysis, the XT_PGX_HuV2_Myeloid CodeSet (NanoString nCounter Human Myeloid Innate Immunity V2 Panel, NanoString technology, Seattle, USA; Supplemental Table 1) was applied according to manufacturer’s protocol. The assay allows the detection of 729 human transcripts and 39 housekeeping genes. In brief, 5 µl of the pooled RNA samples (corresponding to 85–100 ng) were cautiously mixed with 3 µl reporter CodeSet, 5 µl of hybridization buffer, and 2 µl capture ProbeSet, and hybridization was done at 65 °C for 22 h. The purification and counting of gene expression levels were performed fully automated with NanoString nCounterPrepStation and Digital Analyzer. Gene expression analysis was done with nSolver Analysis Software 4.0.

### RT-PCR

cDNAs were prepared with the iScript cDNA Synthesis Kit (Bio-Rad, Germany) and real-time polymerase chain reactions were performed with QuantiTect Primer Assay (Qiagen, Germany) for gene expression of glyceraldehyde-3-phosphate dehydrogenase (GAPDH), collagen type I α2-chain (COL1A2), collagen type XII α1-chain (COL12A1), fibronectin 1 (FN1), leucine-rich repeat containing G protein-coupled receptor 5 (LGR5), serpine peptidase inhibitor member 1 (SERPINE1), and mucin 2 (MUC2). Analysis was done with Bio-Rad CFX-Manager and fold changes calculated with ΔΔC_t_ method.

### RT^2^ profiler pcr arrays

The RNAs of organoids of seven healthy individuals, three patients with active CD, and five patients in remission were pooled, respectively. From each group circa 400 ng of combined RNA was used to prepare the corresponding cDNA with the RT^2^ First Strand Kit (Qiagen, Germany) and further processed with the “human epithelial to mesenchymal transition” PCR array (Qiagen, Germany) according manufacturer’s protocol on a 96-well plate with CFX Connect Real time system (Bio-Rad, Germany). Data analysis was performed using the webportal of Qiagen and fold changes of genes were calculated with the ΔΔC_t_ method with normalization to housekeeping genes. In addition, the RT^2^ profiler PCR array for “human epigenetic chromatin remodeling” (Qiagen, Germany) was done with pooled RNA from the healthy individuals and celiac patients in remission.

## Supplementary information

Dataset 1.
