# Supplementary material for: Intestinal ex vivo organoid culture reveals altered programmed crypt stem cells in patients with celiac disease
Source: Sci Rep. 2020 Feb 26;10:3535. doi: 10.1038/s41598-020-60521-5 (PMC7044285; doi:10.1038/s41598-020-60521-5)
Supplement: Supplementary file 1 — Dataset 1. [file 41598_2020_60521_MOESM1_ESM.docx]

**Title:** “Intestinal ex vivo organoid culture reveals altered programmed crypt stem cells in patients with celiac disease” by Walburga Dieterich, Markus F. Neurath, and Yurdagül Zopf

**Supplemental Figure 1**


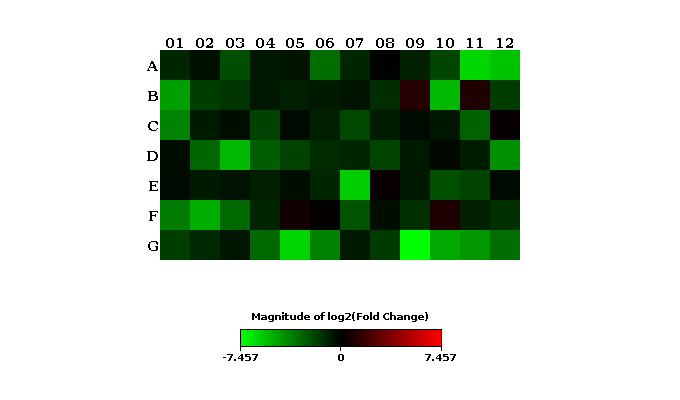

**Supplemental Figure 1:** Gene expression of intestinal organoids generated from patients with CD in remission compared to healthy controls. Heat map shows fold-change expression. Genes under-expressed in organoids from patients with CD in remission (CD gfd; n=5) versus healthy controls (n=7) are shown in green, and corresponding data are listed in the table below. Data analysis was performed using the webportal of Qiagen. Fold changes of genes were calculated with the ΔΔC_t_ method and shown in the corresponding table. Comment A: The gene’s average threshold cycle is relatively high in either the control or the test group and is reasonably low in the other group. Therefore, actual fold-change value is at least as large as the calculated and reported fold-change result. B: The relative gene expression level is relatively low, in both groups. C: Gene expression is very low, making this fold-change result un-interpretable.

**Supplemental Figure 2**


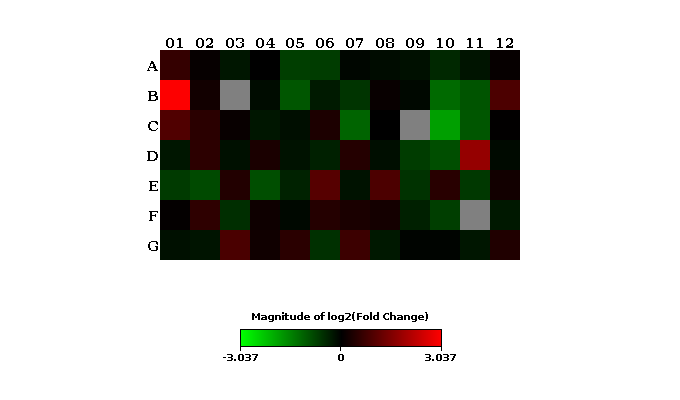

**Supplemental Figure 2:** Gene expression profile to determine chromatin remodeling of organoids from patients with CD compared to healthy controls. Genes over-expressed in organoids from patients with CD (n=2) versus healthy controls (n=2) are shown in red, and corresponding data are listed in the table below. Data analysis was done with webportal of Qiagen, and fold changes calculated with the ΔΔC_t_ method. Patients with CD show upregulation of bromodomain containing 7 (BRD7, 8.21x), inhibitor of growth family member 4 (ING4, 3.46x), and methyl CpG binding protein 2 (MECP2, 2.05x), and decreased expression of chromobox homolog 4 and 5 (CBX4, -2.41x; CBX5, -2.02), chromodomain helicase DNA binding protein 3, 6, and 7 (CHD3, -2.29; CHD6, -3.76; CHD7, -2.02). Comment A: Actual fold-change value is at least as large as the calculated and reported fold-change result. B: The relative gene expression level is relatively low, in both groups. C: Gene expression is very low, making this fold-change result un-interpretable.

| **Suppl Table 1:** Gene expression of organoids generated from duodenal biopsies of healthy controls, patients with active CD, and in remission (CD gfd) | | | | | | | | | |
| --- | --- | --- | --- | --- | --- | --- | --- | --- | --- |
| Gene Name | Accession # | annotations | normalized counts | normalized counts | normalized counts |  |  | fold change | fold change |
|  |  |  | **controls** | **CD activ** | **CD gfd** |  | **controls** | **CD vs control** | **CD gfd vs control** |
| CDH11 | NM_001797.2 | cell migration and adhesion | 22,35 | 1 | 6,61 |  | 1 | -22,8 | -3,38 |
| COL12A1 | NM_004370.5 | ECM remodeling | 239,66 | 4,33 | 4,41 |  | 1 | -55,33 | -54,37 |
| COL1A2 | NM_000089.3 | ECM remodeling | 183,78 | 1,44 | 2,2 |  | 1 | -127,29 | -83,38 |
| COL3A1 | NM_000090.3 | ECM remodeling | 276,91 | 7,94 | 3,31 |  | 1 | -34,87 | -83,76 |
| FGF7 | NM_002009.3 | angiogenesis | 32,29 | 1,44 | 4,41 |  | 1 | -22,36 | -7,32 |
| FGFR1 | NM_015850.3 | angiogenesis | 76,99 | 19,49 | 4,41 |  | 1 | -3,95 | -17,46 |
| FN1 | NM_212482.1 | ECM remodeling | 1154,83 | 17,32 | 20,94 |  | 1 | -66,66 | -55,15 |
| MRC2 | NM_006039.3 | antigen presentation | 68,3 | 2,89 | 3,31 |  | 1 | -23,65 | -20,66 |
| SERPINE1 | NM_000602.4 | ECM remodeling | 444,55 | 2,89 | 6,61 |  | 1 | -153,96 | -67,23 |
| TIMP3 | NM_000362.4 | ECM remodeling | 52,15 | 3,61 | 1,1 |  | 1 | -14,45 | -47,32 |
| TNC | NM_002160.3 | ECM remodeling | 68,3 | 2,17 | 1,1 |  | 1 | -31,54 | -61,97 |
| VEGFC | NM_005429.2 | angiogenesis | 70,78 | 1,44 | 3,31 |  | 1 | -49,03 | -21,41 |
| BCL2 | NM_000657.2 | cell cycle and apoptosis | 3,73 | 1,44 | 4,41 |  | 1 | -2,58 | 1,18 |
| BID | NM_197966.2 | cell cycle and apoptosis | 281,88 | 281,53 | 272,21 |  | 1 | -1 | -1,04 |
| BTG2 | NM_006763.2 | cell cycle and apoptosis | 144,04 | 154,48 | 192,86 |  | 1 | 1,07 | 1,34 |
| CASP8 | NM_001228.4 | cell cycle and apoptosis | 1,24 | 1 | 1,1 |  | 1 | -1,27 | -1,13 |
| CEBPA | NM_004364.2 | cell cycle and apoptosis | 50,91 | 125,6 | 137,76 |  | 1 | 2,47 | 2,71 |
| CEBPD | NM_005195.3 | cell cycle and apoptosis | 298,02 | 279,36 | 329,51 |  | 1 | -1,07 | 1,11 |
| CEBPG | NM_001806.2 | cell cycle and apoptosis | 379,98 | 410,74 | 429,8 |  | 1 | 1,08 | 1,13 |
| HIST1H1C | NM_005319.3 | cell cycle and apoptosis | 1138,69 | 1271,93 | 1151,65 |  | 1 | 1,12 | 1,01 |
| HIST2H2AA3 | NM_003516.2 | cell cycle and apoptosis | 254,56 | 231 | 234,74 |  | 1 | -1,1 | -1,08 |
| ID1 | NM_002165.2 | cell cycle and apoptosis | 2076,21 | 2080,41 | 2288,97 |  | 1 | 1 | 1,1 |
| KIAA0101 | NM_014736.5 | cell cycle and apoptosis | 7,45 | 6,5 | 7,71 |  | 1 | -1,15 | 1,04 |
| MCM5 | NM_006739.3 | cell cycle and apoptosis | 11,18 | 18,77 | 12,12 |  | 1 | 1,68 | 1,08 |
| NDC80 | NM_006101.2 | cell cycle and apoptosis | 22,35 | 22,38 | 13,22 |  | 1 | 1 | -1,69 |
| RGS6 | NM_001204420.1 | cell cycle and apoptosis | 2,48 | 1,44 | 1,1 |  | 1 | -1,72 | -2,25 |
| RHOC | NM_175744.4 | cell cycle and apoptosis | 1667,67 | 1587,38 | 1640,96 |  | 1 | -1,05 | -1,02 |
| RHOG | NM_001665.3 | cell cycle and apoptosis | 119,21 | 124,16 | 121,23 |  | 1 | 1,04 | 1,02 |
| RHOJ | NM_020663.4 | cell cycle and apoptosis | 1,24 | 2,17 | 1,1 |  | 1 | 1,74 | -1,13 |
| RND3 | NM_005168.3 | cell cycle and apoptosis | 89,41 | 51,25 | 72,74 |  | 1 | -1,74 | -1,23 |
| STK17A | NM_004760.2 | cell cycle and apoptosis | 325,34 | 298,13 | 285,43 |  | 1 | -1,09 | -1,14 |
| TERF2IP | NM_018975.2 | cell cycle and apoptosis | 125,42 | 114,78 | 155,39 |  | 1 | -1,09 | 1,24 |
| TFCP2 | NM_005653.3 | cell cycle and apoptosis | 111,76 | 103,95 | 115,72 |  | 1 | -1,08 | 1,04 |
| VRK2 | NM_006296.3 | cell cycle and apoptosis | 62,09 | 59,91 | 72,74 |  | 1 | -1,04 | 1,17 |
| ABCF1 | NM_001090.2 | House keeping | 187,5 | 189,13 | 192,86 |  | 1 | 1,01 | 1,03 |
| AGK | NM_018238.3 | House keeping | 44,7 | 49,81 | 38,57 |  | 1 | 1,11 | -1,16 |
| ALAS1 | NM_000688.4 | House keeping | 34,77 | 49,81 | 49,59 |  | 1 | 1,43 | 1,43 |
| AMMECR1L | NM_031445.2 | House keeping | 80,71 | 65,69 | 82,65 |  | 1 | -1,23 | 1,02 |
| CC2D1B | NM_032449.2 | House keeping | 103,07 | 97,45 | 88,16 |  | 1 | -1,06 | -1,17 |
| CNOT10 | NM_001256741.1 | House keeping | 55,88 | 54,86 | 60,61 |  | 1 | -1,02 | 1,08 |
| CNOT4 | NM_001190848.1 | House keeping | 58,36 | 56,31 | 63,92 |  | 1 | -1,04 | 1,1 |
| COG7 | NM_153603.3 | House keeping | 103,07 | 90,23 | 137,76 |  | 1 | -1,14 | 1,34 |
| DDX50 | NM_024045.1 | House keeping | 59,6 | 60,64 | 49,59 |  | 1 | 1,02 | -1,2 |
| DHX16 | NM_001164239.1 | House keeping | 75,75 | 82,29 | 71,63 |  | 1 | 1,09 | -1,06 |
| DNAJC14 | NM_032364.5 | House keeping | 12,42 | 23,1 | 16,53 |  | 1 | 1,86 | 1,33 |
| EDC3 | NM_001142443.1 | House keeping | 81,96 | 83,74 | 101,39 |  | 1 | 1,02 | 1,24 |
| EIF2B4 | NM_172195.3 | House keeping | 117,97 | 102,5 | 121,23 |  | 1 | -1,15 | 1,03 |
| ERCC3 | NM_000122.1 | House keeping | 43,46 | 28,15 | 26,45 |  | 1 | -1,54 | -1,64 |
| FCF1 | NM_015962.4 | House keeping | 150,25 | 131,38 | 197,27 |  | 1 | -1,14 | 1,31 |
| G6PD | NM_000402.2 | House keeping | 139,08 | 118,39 | 146,57 |  | 1 | -1,17 | 1,05 |
| GPATCH3 | NM_022078.2 | House keeping | 17,38 | 19,49 | 13,22 |  | 1 | 1,12 | -1,31 |
| GUSB | NM_000181.3 | House keeping | 327,82 | 282,97 | 290,94 |  | 1 | -1,16 | -1,13 |
| HDAC3 | NM_003883.3 | House keeping | 155,22 | 153,04 | 174,12 |  | 1 | -1,01 | 1,12 |
| HPRT1 | NM_000194.1 | House keeping | 270,7 | 246,88 | 283,23 |  | 1 | -1,1 | 1,05 |
| MRPS5 | NM_031902.3 | House keeping | 197,44 | 194,9 | 192,86 |  | 1 | -1,01 | -1,02 |
| MTMR14 | NM_022485.3 | House keeping | 161,43 | 153,76 | 135,55 |  | 1 | -1,05 | -1,19 |
| NOL7 | NM_016167.3 | House keeping | 319,13 | 327,73 | 340,53 |  | 1 | 1,03 | 1,07 |
| NUBP1 | NM_002484.3 | House keeping | 103,07 | 131,38 | 156,49 |  | 1 | 1,27 | 1,52 |
| POLR2A | NM_000937.2 | House keeping | 177,57 | 170,36 | 188,45 |  | 1 | -1,04 | 1,06 |
| PPIA | NM_021130.3 | House keeping | 4442,99 | 5354,79 | 5833,17 |  | 1 | 1,21 | 1,31 |
| PRPF38A | NM_032864.3 | House keeping | 168,88 | 165,31 | 128,94 |  | 1 | -1,02 | -1,31 |
| SAP130 | NM_024545.3 | House keeping | 124,18 | 122,72 | 131,14 |  | 1 | -1,01 | 1,06 |
| SDHA | NM_004168.1 | House keeping | 249,59 | 284,41 | 243,55 |  | 1 | 1,14 | -1,02 |
| SF3A3 | NM_006802.2 | House keeping | 96,86 | 60,64 | 79,35 |  | 1 | -1,6 | -1,22 |
| TBP | NM_001172085.1 | House keeping | 53,4 | 51,97 | 39,67 |  | 1 | -1,03 | -1,35 |
| TLK2 | XM_011524223.1 | House keeping | 132,87 | 144,37 | 134,45 |  | 1 | 1,09 | 1,01 |
| TMUB2 | NM_024107.2 | House keeping | 100,58 | 85,9 | 87,06 |  | 1 | -1,17 | -1,16 |
| TRIM39 | NM_021253.3 | House keeping | 21,11 | 22,38 | 16,53 |  | 1 | 1,06 | -1,28 |
| TUBB | NM_178014.2 | House keeping | 422,2 | 325,56 | 310,78 |  | 1 | -1,3 | -1,36 |
| USP39 | NM_001256725.1 | House keeping | 93,13 | 90,23 | 111,31 |  | 1 | -1,03 | 1,2 |
| ZC3H14 | NM_207662.3 | House keeping | 146,53 | 192,74 | 157,59 |  | 1 | 1,32 | 1,08 |
| ZKSCAN5 | NM_014569.3 | House keeping | 14,9 | 15,16 | 9,92 |  | 1 | 1,02 | -1,5 |
| ZNF143 | NM_003442.5 | House keeping | 48,43 | 56,31 | 65,02 |  | 1 | 1,16 | 1,34 |
| ZNF346 | NM_012279.3 | House keeping | 6,21 | 7,22 | 7,71 |  | 1 | 1,16 | 1,24 |

**Supplemental Table 1:** Gene expression of organoids generated from duodenal biopsies of healthy controls (n=5), patients with active CD (n=2), and in remission (CD gfd; n=5) determined by NanoString Human Myeloid Innate Immunity V2 Panel. Shown are normalized data of most differently expressed genes, genes associated with cell cycle and apoptosis, as well as housekeeping genes from all groups and fold change of CD and CD gfd related to healthy control data.

| **Suppl Table 2**: Changing culture conditions causes differentiation of organoids | | | | |
| --- | --- | --- | --- | --- |
|  | **LGR5** | | **Muc2** | |
|  | patient 1 | patient 2 | patient 1 | patient 2 |
| CM-S | 1 | 1 | 1 | 1 |
| Differentiation medium | 0,206 | 0,0058 | 3,719 | 62,043 |

**Supplemental Table 2:** Deprivation of growth factors and supplementation of culture medium with γ-secretase inhibitor DAPT cause differentiation of intestinal organoids. Exemplary relative gene expression of stem cell marker LGR5 and goblet cell marker mucin 2 (Muc2) from organoids of patients with non-celiac glutensensitivity under normal culture medium (CM-S) and after 2 days of differentiation is shown. Differentiation is confirmed by downregulation of LGR5 and upregulation of Muc2.

**Supplemental Table 3:** RT-PCR amplification of genes of interest from single individuals. Increased expression is labelled in grey. Fold-change analysis was done with ΔΔC_t_ method and values were correlated to basic expression of sample from celiac patient on gfd (*). GAPDH was used for housekeeping gene.

**Supplemental Table 4:** Influence of culture medium on ECM gene expression. RT-PCR amplification of COL12A1, FN1, and SERPINE1 with pooled cDNA from organoids of controls and celiac patients on gfd (n=3, respectively). Fold-change analysis was done with ΔΔC_t_ method and values were correlated to basic expression of pooled cDNA from CD patients on gfd, with GAPDH for housekeeping gene. Non-differentiated organoids on CM-S culture medium showed no different gene expression. Changing culture medium (Diff) resulted in increased ECM gene expression of organoids from healthy controls compared to organoids from patients with CD on gfd.

**Supplemental Table 5:** RT-PCR amplification of FN1 and SERPINE1 from organoids of three healthy individuals and patients with CD on gfd (n=3, respectively). Fold-change analysis was done with ΔΔC_t_ method and values were correlated to basic expression of CD patient on gfd. GAPDH was used for housekeeping gene. Non-differentiated organoids on standard CM-S culture medium showed no different gene expression. Changing culture medium (Diff) resulted in increased ECM gene expression of organoids from two healthy controls compared to organoids from patients with CD on gfd. The control who showed no increased ECM values revealed activated lymph follicles in histopathology, and thus rather must be classified as a sick individual.

**Supplemental Table 6:** RT-PCR with cDNA from duodenal biopsy samples. Fold-changes of COL1A2, FN1, and SERPINE1 were calculated with the ΔΔC_t_ method. GAPDH was used for housekeeping gene and data were normalized and correlated with patient with CD under remission (*). No differential gene expression was noticed in intestinal biopsies from healthy controls (n=3), patients with active CD (n=2) or patients on gfd (CD gfd; n=5).

**Supplemental Table 7:** Genes over-expressed in organoids derived from healthy controls (n=7) versus patients with active CD (n=3) or under remission (CD gfd; n=5). Data was generated using the RT^2^ Profiler PCR Arrays gene array for human epithelial to mesenchymal transition (Qiagen, Germany)**.** Fold-changes of genes were calculated with the ΔΔC_t_ method, and GAPDH was used for housekeeping gene. Fold-changes >2 are marked in grey.
